# Analysing food farming vulnerability in Kalimantan, Indonesia: Determinant factors and adaptation measures

**DOI:** 10.1371/journal.pone.0296262

**Published:** 2024-01-03

**Authors:** Woro Estiningtyas, Elza Surmaini, Suciantini, Erni Susanti, Anny Mulyani, Budi Kartiwa, Sumaryanto, Perdinan, Yayan Apriyana, Annisa Dhienar Alifia

**Affiliations:** 1 Research Center for Climate and Atmosphere, National Research and Innovation Agency, Bandung, Indonesia; 2 Research Center for Food Crop, National Research and Innovation Agency, Bandung, Indonesia; 3 Research Center for Limnology and Water Resources, National Research and Innovation Agency, Bandung, Indonesia; 4 Research Center for Behavioral and Circular Economics, National Research and Innovation Agency, Bandung, Indonesia; 5 Faculty of Mathematics and Natural Science, IPB University, Bogor, Indonesia; 6 Research Centre for Horticultural and Estate Crops, National Research and Innovation Agency, Bandung, Indonesia; University of Glasgow College of Medical Veterinary and Life Sciences, UNITED KINGDOM

## Abstract

As a result of plans to relocate the Indonesian capital city to East Kalimantan province, Kalimantan is expected to develop rapidly and the surrounding regencies and provinces will become food support areas for the new capital. However, the vulnerability of food farming in Kalimantan is a concern that must be addressed to ensure food security. This study aims to assess the vulnerability of food farming at the regency level of the island of Kalimantan, to assess the determinant factors of the food farming vulnerability and to compose adaptation measures that can reduce vulnerability. Socio economic, climate, water and land data are sorted and analyzed to represent the level of sensitivity and exposure index (SEI) and adaptive capacity index (ACI). Locations with ‘High’ and ‘Very High’ levels of farming vulnerability become interview sites with a total of 150 respondents. The results of the interviews strengthen the results of the vulnerability analysis which helps to determine the condition of farmers and food farming in vulnerable locations. The results indicated ‘Very High’ and ‘High’ level of vulnerability in 14 regencies/cities. Floods are climate-related disasters that most often affect farmers surveyed (46%), followed by droughts (30%) and pest attacks (24%) with significant impacts (49%). The identification of the determinant factors becomes the basis for adaptive measures to support decision-makers, local practitioners, and farmers by highlighting local challenges and proposing local-specific adaptation strategies.

## Introduction

Agriculture in developing countries faces a notable significant from climate change [1, 2]. Several studies have shown that climate change has a negative impact on agricultural production due to the emergence of pest attacks, increased incidence of floods and droughts, crop failures and livestock mortality [3–6]. In developing countries, smallholder farmers are among the social groups highly susceptible to the impacts of climate change [7], particularly in rural communities whose livelihoods rely upon small-scale agriculture [3, 8], which has a severe negative impact on their household well-being and food security [9–11].

Climate change has implication for food production, farmers’ income, food accessibility, food supply, and food security [12–14]. Indonesia is considered prone to the impacts of climate change because of political, geographical, and social factors [15], including extreme events such as floods and droughts, long-term changes in sea level rise, changes in rainfall patterns, and increases in temperature [15]. Rice, as the staple food of the Indonesian people, is heavily affected by climate change [16–18] and climate variability [19, 20]. Furthermore, rice production is vulnerable to changes in the onset and duration of the wet season. A 30-day monsoon delay resulted in rice production falling by an average of 11% in East Java/Bali and 6.5% in West/Central Java [17]. El Niño events influence rice production, delaying rainfall and increasing the risk of annual rice deficits [15, 20–23].

Vulnerability is a function of three dimensions: Exposure to hazards, Sensitivity to damage, and Adaptive Capacity. Exposure appertains to the extent and characteristics of a system exposed to significant climate variability. Sensitivity is the influence degree as a system stimulated by climatic factors. While adaptive capacity refers to the ability to project and avoid losses regarding climate change’s adverse impact on natural and man-made systems [24]. Therefore, the aggregation of these elements is conceptualized as the climate change impact on agriculture.or when a system experiences stress due to pressure [25]. Vulnerability assessment is a useful planning tool in developing a climate-appropriate sector adaptation strategy. The number of vulnerability assessment from scientific literature focusing on various sectors, including agriculture [26–28]. Vulnerability assessments can assist in identifying which communities are most vulnerable to climate change, their location, and the causes of their vulnerability [29]. Understanding the potential impacts lets decision-makers make objective guidance for adaptation planning, publicize climate change policies and for accessing climate finance [30–32].

The evaluation of vulnerability can be conducted at various levels, including regional or national scales, sub-national, communities, and even households or individuals [32–34]. The methodology employed is based on the research objectives and geographical scope of the study [35]. The primary objective of the study in this context is agriculture. Agriculture is typically influenced by two key groups, which are natural factors and socio-economic factors as input indicators [36, 37]. To achieve this, it is crucial to identify the indicators that make food farming vulnerable for the food support areas in the new capital, which is to increase food production and reduce yield losses.

The Indonesian government has announced the relocation of the new capital of Indonesia from Jakarta to the province of East Kalimantan which will be in the regencies of Penajam Paser Utara and Kutai Kartanegara. Building a city on the forested Kalimantan Island will result in land conversion including agricultural land, and could increase the vulnerability of the food farming system. To ensure an adequate food supply for the growing population of the new capital, certain surrounding regencies such as Berau, Tanah Bumbu, Bulungan, and Nunukan in East Kalimantan Province, and some food producing regencies in South Kalimantan, Central Kalimantan and West Kalimantan will serve as food support areas for the new capital. If left unaddressed, climate change and extreme weather conditions will pose major challenges for agricultural growth in Kalimantan, particularly for food crops [38, 39].

Agricultural land in Kalimantan Island comprises both wetland and dry land. Wetlands are composed of tidal swamps and lowland swamps that contain peat. Due to the absence of volcanoes, the vast dry land on this island is dominated by non-volcanic land. These two lands have low and medium fertility levels, resulting in them being less fertile than other Indonesian islands. Given these land characteristics, it is reasonable to infer that the soil fertility index plays a crucial role in influencing the productivity of food crops in Kalimantan. In addition, over the last 30 years, Kalimantan has generally experienced an increased in rainfall, particularly in regions with annual rainfall regimes, while semi-annual rainfall regimes have become drier. Moreover, the dry and wet seasons begin and end one month earlier than usual [38]. Furthermore, floods and droughts are becoming more frequent in several regencies of Kalimantan [39].

Studies on food farming vulnerability in Indonesia at the regency level are limited and employed various indicators. Takama et al. [40] assess rice vulnerability in Bali Province using indicators such as drought, land use change, water level, land and price. Arifah et al. [41] focused on the vulnerability of irrigation access in South Sulawesi, while Suryanto et al. [42] and Murniati & Mutolib [43] incorporated socio-economic indicators such as income, consumption habits and education levels. The selection of more relevant indicators to develop a vulnerability assessment for food farming in Kalimantan Island is what distinguished this research.

Socio-economic indicators are also important to consider in this study. Moreover, given that the area is the new capital, with its rapidly growing population and the conversion of agricultural land into infrastructure, these changes will inherently have an effect on income, food demand, pricing, and diversification. Consequently, we consider the Gross Regional Domestic Product (GRDP), the Gini index, the proportion of the population in poverty, food expenses, and entropy as indicators that best encapsulate these circumstances. The objectives of this study are threefold: first, to assess food farming vulnerability at the regency level in Kalimantan Island; second, to assess the determinant factors of food farming vulnerability; and third, to compose adaptation measures to reduce the vulnerability of food farming.

## Material and method

### Study area

**Fig 1:**
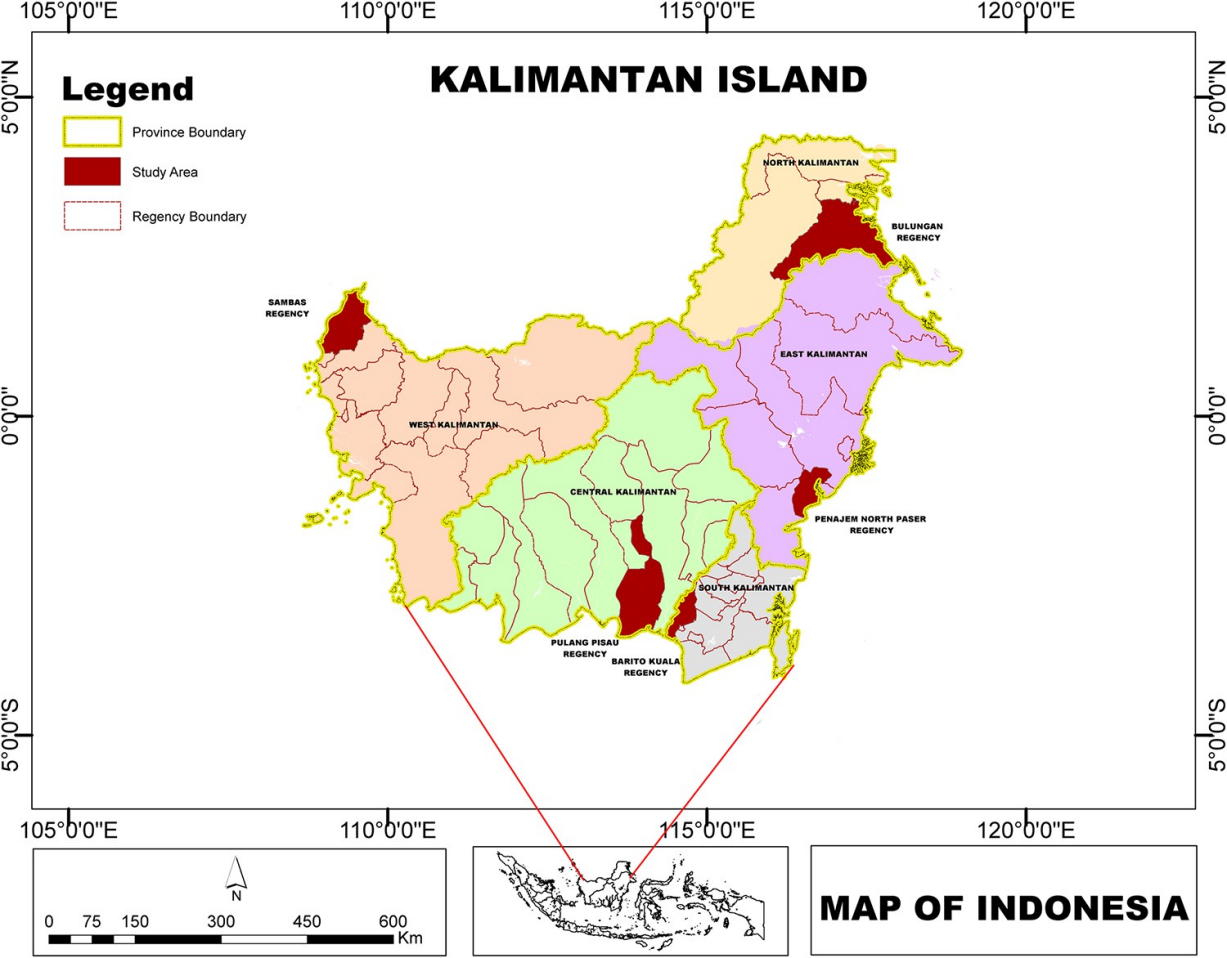
The study area encompasses all the regencies in Kalimantan, and the interview location (shaded area).

Kalimantan is the southern three-quarters part of the Borneo Island, located in Indonesia and accounts for 73% of the island’s surface area. It is situated in the geographic center of the Maritime Continent, situated north of Java, west of Sulawesi, and east of Sumatra. There are five provinces in Kalimantan: South Kalimantan, East Kalimantan, Central Kalimantan, West Kalimantan, and North Kalimantan (Fig 1). The mean annual rainfall within the five provinces indicates that Kalimantan receives rainfall ranging between 2000–3300 mm/year, with 200–240 rainy days/year and mean air temperature of 27.1 and 28.7°C and mean humidity of 79–85%. Agriculture is the primary source of livelihood, followed by forestry, fisheries and trade.

Kalimantan covers an area of 52.3 million ha, of which the majority (77.8%) or about 40.7 million ha, is predominantly dry land dominated by Ultisols and Oxisols soils. The soils have undergone further development and leaching of nutrients resulting in a low soil fertility level. Furthermore, approximately 7.7 million ha of the 40.7 million ha are hilly areas (slopes between 15–25%) and 12.3 million ha are mountains with slopes exceeding 25%, which are at risk from the dangers of erosion and degradation. The wetlands, covering an area of 11.6 million ha are comprised of peatlands (Haplohemist 4.3 million ha), Endoaqupis 3.6 million ha, and the remaining are other swamplands (Hydraquents and Sulfaquepts) which are not suitable for agricultural development. The area of Kalimantan Island that can be utilised for agriculture is broken down into 6.9 million ha for plantations, 2.2 million ha for seasonal dry land farming, and 0.7 million ha for rice fields.

To assess the vulnerability of rice cultivation in the island of Kalimantan, all regencies in the five provinces were evaluated. However, to gather information on risks related to agriculture, interviews were conducted in a single regency in each province, selected to represent a very high or high degree of vulnerability. The five regencies selected for the interview were Sambas of West Kalimantan, Pulang Pisau of Central Kalimantan, Barito Kuala of South Kalimantan, Tanjung Palas of North Kalimantan and North Panajam Paser of East Kalimantan. These regencies have been selected because they are vulnerable to climate change impacts on food farming. The information gathered through these interviews will develop recommendations to reduce vulnerability and increase the resilience of food farming in these regencies and across the region.

### Materials

The assessment of food vulnerability is based on indicators that focus on soils, climate, water resources and socioeconomic aspects. Indicators were determined by expert judgement through a focus group discussion. Official data sources have been used to compute vulnerability indicators. All data used for the analysis of rice vulnerability indicators were collected and presented in Table 1. The attributes presented on the resulting maps (Figs 1, 5, and 6) are complemented by the administration map (Statistics Indonesia 2015) and the base map (Geospatial Information Agency 2018).

**Table 1:** Data types and sources used in this study.

Data	Source
Soil Reconnaissance Map (1:250,000)	Indonesian Centre for Agricultural Land Resources Research and Development (ICALRRD 2014)
Administration Map (1: 250,000)	Statistics Indonesia (2015)
Basemap (1:50,000)	Geospatial Information Agency 2018
Map of official rice area (1:5,000)	Centre for Data and Information System-Ministry of Agriculture
Monthly Rainfall data	Meteorological, Climatological, and Geophysical Agency, Ministry of Public Work, Indonesian Agro-Climate and Hydrology Institute (IAHRI) database (2018)
Water Discharge	Ministry of Public Work (2018)
Type and area of irrigation	Statistics Indonesia (2015)
Number of reservoirs per area	Ministry of Public Work (2018)
Length of irrigation network per area	Ministry of Public Work (2018)
Crop production	Statistics Indonesia (2015)
Land area	Statistics Indonesia (2015)
Harvested area	Statistics Indonesia (2015)
Farmer household	National Socio-Economics Survey Data, Statistics Indonesia (2015)
School participation rate	Statistics Indonesia (2015)
Road length based on surface condition	Statistics Indonesia (2015)
Number of extension workers	Centre for Data and Information System-Ministry of Agriculture (2018)
Number of farmers groups	Centre for Data and Information System-Ministry of Agriculture (2018)
Number and types of agricultural machinery	Integrated Cropping Calendar version 2.4 (2018)
Food consumption	National Socio-Economics Survey Data, Statistics Indonesia (2015)
Food expenditure	National Socio-Economics Survey Data, Statistics Indonesia (2015)
Percentage of poor people	Statistics Indonesia (2015)
Gross Regional Domestic Product (GRDP)	Statistics Indonesia (2015)
Gini index	National Socio-Economics Survey Data, Statistics Indonesia (2015)
Agroclimatic type	Oldeman (1980)
Population density	Statistics Indonesia (2015)

## Methodology

### Conceptual framework

**Fig 2:**
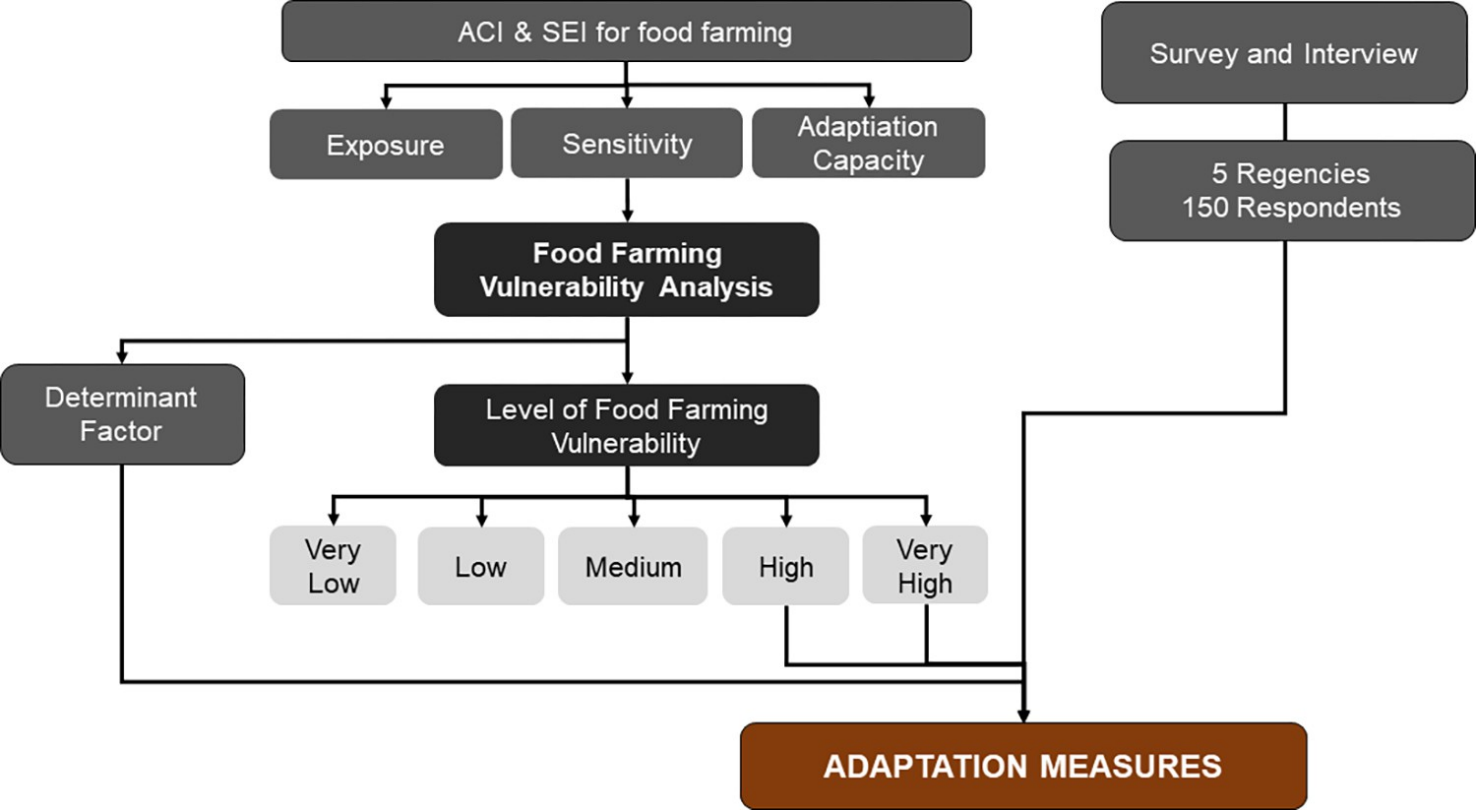
Illustration of the relationship between activities in the assessment of food farming vulnerability.

Food availability can be improved by developing Kalimantan Island, which covers more than a quarter of Indonesia’s area. The people of Kalimantan who rely on agriculture for their livelihood and consume rice as a staple food, requires assurances for the sustainability of their food farming. The food farming vulnerability study provides information on the regency vulnerability level in Kalimantan Island that can be utilised to develop adaptation efforts. The 2012 IPCC concept is used to analyse the vulnerability of food farming based on secondary data of the regency. ACI and SEI indicators were selected due to their relevance to food farming and the availability of necessary data from various sources at the regency level throughout the island of Kalimantan. The vulnerability level resulting from this study is the vulnerability at the regency level. This means that the vulnerability assessment was conducted for each regency on Kalimantan Island. To identify the current conditions of food farming related to the vulnerability of food farming, regencies with a vulnerability level of "High" and "Very High" were selected for interviews to obtain information about: 1) farmers characteristics, 2) climate-related disasters, impacts and farmers responses, 3) farming characteristics, and 4) farmers challenges and expectations in food farming. Adapted measures are composed based on the results of surveys and interviews, as well as determining factors (Fig 2).

**Fig 3:**
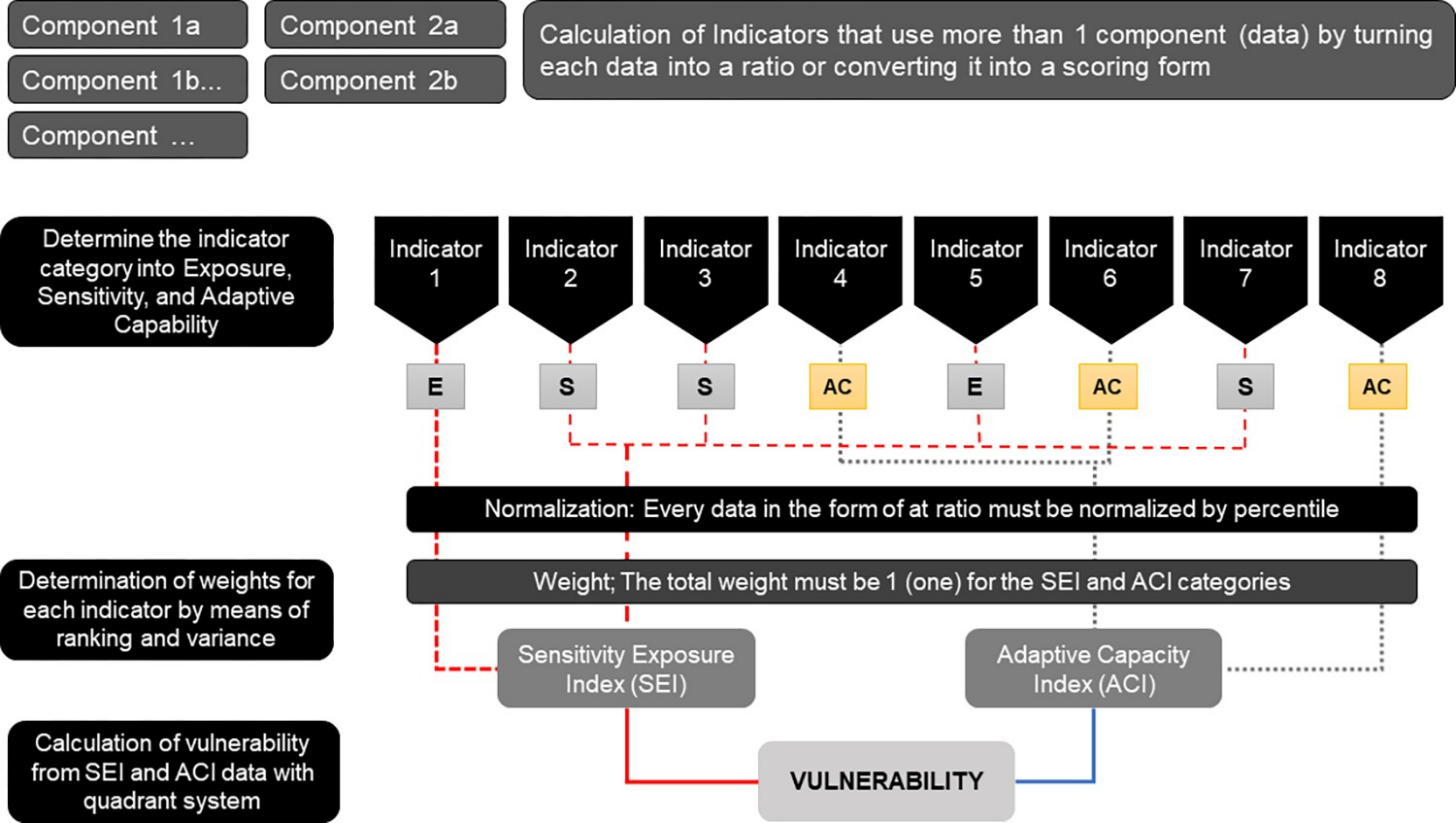
Stages in analysing the vulnerability of food farming.

Fig 3 describes the vulnerability analysis process of the regencies in the island of Kalimantan. The first step consisted of selecting a proper indicator relevant to the vulnerability of food farming. The indicators were grouped based on the Adaptive Capacity Index (ACI) and Sensitivity Exposure Index (SEI). Both ACI and SEI were represented by 15 and 5 indicators respectively. The definition and equation of ACI and SEI are presented in Table 2. Soil fertility and climate indexes are detailed in the following paragraph.

**Table 2:** Definition and equation of Adaptive Capacity Index (ACI), Sensitivity Exposure Index (SEI), and indicator’s relationship to vulnerability.

Indicators	Definition	Equation	Relationship to vulnerability
**Adaptive Capacity Index (ACI)**			
ACI 1	School Participation Rate	${ACI}_{1}=\sum_{i}^{n}w_{i}\times {AC}_{1i}$ *I* = primary school, junior high school, senior high school w_i_ = 0.2, 0.3, 0.5	The higher the participation rate, the greater the adaptive capacity.
ACI 2	Road length based on surface conditions	${ACI}_{2}=\frac{\sum_{i}^{n}w_{i}\times {AC}_{2i}}{Area}$ *i* = asphalt, gravel, soil, concrete, paving block, lapen, not specified w_i_ = 0.21,0.05,0.26 0.11,0.16,0.05	The distribution of goods and services is improved by improving the quality of transportation, which supports increased adaptive capacity.
ACI 3	Ratio of the number of extension officers to rice field area	${\;ACI}_{3}=\frac{Number\;of\;extention\;officer}{Rice\;field\;area}$	The increased in the number of trained extension officers indicates conducive extension activities that positively contributes to rice production.
ACI 4	Ratio of the number of farmer groups to rice field area	${\;ACI}_{4}=\frac{Number\;of\;farmer\;groups}{Rice\;Fiels\;area}$	The growing number of skilled and empowered farmer groups will ensure the efficiency of extension activities, leading to a greater adaptive capacity.
ACI 5	Ratio of the number of agricultural machineries to rice field area	${ACI}_{5}=\frac{Number\;of\;agriculture\;machinery}{Rice\;field\;area}$	The expansion of rice farming areas is hindered by the insufficient availability of agricultural machinery. Enhancing adaptive capacity can be achieved by increasing the ratio of agricultural machinery to rice field area.
**Sensitivity Exposure Index (SEI)**			
SEI 1	Ratio of rice consumption to total carbohydrate food	${SEI}_{1}=\frac{Rice\;Consumption}{Total\;carbohydrate}$	Food security becomes vulnerable due to a higher dependency on rice. Advancing non-rice local food technology production can result in a decreased in the ratio of rice consumption to total carbohydrates, which can decrease sensitivity and exposure indices.
SEI 2	Rice consumption per capita	Statistical data	Reducing the ratio of rice consumption per capita serves as a viable option for lowering sensitivity and exposure indices.
SEI 3	Entropy (food diversification level)	${\;SEI}_{3}=-\sum_{i=1}^{n}\{((\frac{ki*Ci}{\sum ki}*Ci)ln((ki*Ci))\}/ln\;k$ SEI_3_ = Entropy index to measure the diversification of carbohydrate foods k_i_ = value of carbohydrate content of food commodity type i. C_i_ = consumption of food commodity sources type i.	A decrease in sensitivity and exposure indices can be caused by a higher level of entropy, which leads to a wider variety of food choices being consumed.
SEI 4	Ratio of expenditure on rice to total expenditure on food	${SEI}_{4}=\frac{Rice\;exp}{total\;\exp\;on\;food\;}$	The Sensitivity and Exposure Index decreases as the ratio of rice expenditure to total food expenditure decreases.
SEI 5	Percentage of poor people	data	Poor people typically have a low adaptive capacity. Therefore, the lower the percentage of poor people, the lower the Sensitivity and Exposure Index.
SEI 6	Ratio of rice and maize production to total population	${SEI}_{6}=\frac{Rice\;Corn}{Population}$	Rice and maize are the primary sources of carbohydrate. The lower the ratio of rice and maize production to total population, the lower the Sensitivity and Exposure Index.
SEI 7	Ratio of soybean production to total population	${SEI}_{7}=\frac{Soybeans}{Population}$	Soybean is the main plant-based protein source for the general Indonesian population (in processed forms like tofu and tempeh).
SEI 8	Water critically index	${SEI}_{8}=\frac{W_{n}}{W_{s}}x\;100\%$ SEI_8_: water critically index (%) W_n_: water demand (m^3^) W_s_: water availability (m^3^)	The lower the water critically index, the lower the Sensitivity and Exposure Index.
SEI 9	Soil fertility level	Percentage of soil fertility and slope	The lower the soil fertility level, the lower the Sensitivity and Exposure Index.
SEI 10	Ratio of agricultural GRDP to total GRDP	${SEI}_{10}=\frac{GRDP\_agr}{total\;GRDP}$	Higher SEI 10 indicates a greater significance of the agricultural sector to the region’s economy. Despite being vulnerable to climate change, the agricultural sector has the most potential for mitigating climate change. The lower the ratio of agricultural GRDP to total GRDP, the lower the Sensitivity and Exposure Index.
SEI 11	Gini Index (income gap)	${SEI}_{11}=1-\sum fi[Yi+Yi-1]$ fi = the number of percent (%) of class i income recipients. Yi = cumulative amount (%) of income in class i	Higher income distribution (lower Gini index) is estimated to be conducive to higher Adaptive Capacity and lower the Sensitivity and Exposure Index.
SEI 12	Climate Index	Classified by climate type based on Oldeman (1979)	The lower the climate index, the more the agricultural efforts are required to adapt to climatic conditions, to further reduce sensitivity and exposure indices.
SEI 13	Ratio of farmer households to total households	${\;SEI}_{13}=\frac{Farmer\;household}{total\;household}$	The proportion of farmer household sensitivity and exposure indices.
SEI 14	Population density (per Km Square)	${SEI}_{14}=\frac{Population}{Area}$	The availability of resources per capita was impacted by changes in population density. A decreased in population density leads to a decrease in sensitivity and exposure indices
SEI15	Ratio of land area for agriculture to total area	${SEI}_{15}=\frac{Land\;area\;for\;agriculture}{Total\;area}$	The higher the population, the less land and water resources available per capita. An increasing in the ratio of agricultural land area to the total area contributes to a decrease in sensitivity and exposure indices.

#### Soil Fertility Index (SEI 9)

Soil fertility was determined using soil names and classifications [44, 45], which were derived from Soil Reconnaissance Maps on a scale of 1: 250,000 [46]. Information on the map includes the name of the soil (soil classification), the landform, the parent material, and the slopes. The parent material greatly determines the soil classification [47], the same parent material can lead to different soil classifications, as well as landform. Soil quality is therefore determined by classifying soils by the major group [48]. Each soil classification implies several soil chemical properties, the level of soil development, and can reflect the level of soil fertility, as well as its potential for agricultural development [49] for both wet and dry lands. For example, Eutrudepts has a base saturation > 50%, whereas Dystrudepts has a base saturation < 50%, meaning that Eutrudepts soil has a higher fertility rate than Dystrudepts as it has higher exchangeable bases. Mollisols soils have Mollic epipedons, some of which have C–organic > 2.5% and base saturation > 50% [48]. The soils of the Orders of Alfisols and Andisols generally have a higher level of soil fertility than those of Ultisols and Oxisols.

Based on the soil classification and the soil properties as mentioned above, all soil types on the island of Kalimantan were grouped into five and given a score from 1 to 5. Soil Ordos which came from volcan parent material and contain highest chemical characteristics and properties were given a score of 5, consisting of a part of Mollisols. Alfisols, Andisols, Vertisols. Score of 4 is given if the soils have ustic moisture regime and other properties in Vertisols and Inceptisols. Most Inceptisols, Ultisols, and Oxisol which have less than 35% base saturation throughout the soil are grouped into score 3. Soil in swamp and peat areas were given a score of 2. The sandy soils, shallow soils, or rock outcrop (ROC) were given a score of 1 (Table 3).

**Table 3:** Classification of soil great group and their scores in Kalimantan Island.

Major group of soil	Score
Epiaquands, Endoaquands, Hapludalfs, Hapludands, Hapluderts, Hapludolls, Haprendolls, Agriudolls, Haplustalfs, Udivitrands, Paleudalfs	5
Endoaquepts, Epiaquepts, Eutrudepts, Haplustepts, Cromusterts, Paleusterts, Kanphaludults, Haplohumults, Haplustalfs, Haplustepts, Paleudalfs,	4
Dystrudepts, Hapludults, Acrudox, Hapludox, Haplustults, Kandiudults, Paleudults, Paleustults, Eutrodox, Inceptisols (ordo), Kandiudox, Udifluvents,	3
Halaquepts, Hydraquents, Sulfaquents, Haplofibrist, Udipsamments, Ustipsamments, Sulfaquepts, Sulfihemist, Sulfisaprist, Placaquods, Psammaquents, Haplosaprist, Haplohemist, Plinthudults, Fluvaquents, Endoaquents,	2
Haplorthods, Fragiorthods, Udorthents, Quartzipsamments, ROC	1

The slope class is regarded as a limiting factor, related to the suitability of the land for agricultural development, in terms of environmental sustainability, erosion hazards and land degradation. Land on very steep slopes (>40%) is classified as unsuitable for agriculture [50] although the soil is fertile, thus it is given a score of 1. Technical guidance of land evaluation [50] slopes less than 8% for paddy fields (a score of 5); 8–15% for other food crops (a score of 4), 15–25% for estate/perennial crop (a score of 3); and 25–40% for certain perennial crop (such as cacao, coffee plant, durians, rambutans, etc.). Accordingly, the slope of the land was divided into five groups as shown in Table 4. With these two factors in mind, an algorithm was developed to determine the soil fertility level (Table 5). The combination of soil and slope scores was used to determine the soil fertility index. High soil score and flat slope score obtained a soil fertility index of 1 (very fertile), while very low soil score and mountain slope score obtained soil fertility index of 0.10 (infertile).

**Table 4:** Classification of slope level and their scores on the island of Kalimantan.

Slope level	Score
Flat to undulating (0–8%)	5 (Very suitable)
Rolling (>8–15%)	4 (Suitable)
Hillock (>15–25%)	3 (Marginally Suitable)
Hilly (>25–40%)	2 (Suitable for Non Food Crop)
Mountainous (> 40%)	1 (Not Suitable)

**Table 5:** Algorithm for determining the index of soil fertility at regency level.

Soil score	Slope score	Soil score x slope score	Total scores at regency level	Soil fertility index	Soil fertility at regency level
5	5	25	400–500	1	Very fertile
4	4	16	300–400	0,75	Fertile
3	3	9	200–300	0,5	Moderately fertile
2	2	4	100–200	0,25	Slightly fertile
1	1	1	< 100	0,10	Not fertile

#### Climate Index (SEI 12)

In determining the climate index, the process begins by establishing rankings based on the Oldeman classification order. Oldeman carried out a classification from A to E3. The ranking is done simply by sorting classes A, B1, B2, C1 and so on up to E3 and resulting in 12 climate types. Considering that type A represents the wettest category, it is assigned the highest ranking of 12, followed by B1 with a ranking of 11, and so forth, with E3 being assigned the lowest ranking. Each climate type is then compared to its maximum value, which is 12. The ranking values derived from this comparison are subsequently utilized to determine the index assessment. Consequently, type A, representing the category with the highest precipitation, obtains the highest index value of 1, trailed by type B at 0.92, and so on, down to type E3 at 0.08 (Table 6).

**Table 6:** Oldeman climate type, rank, and score.

Climate Type	Rank	Score
A	12	1
B1	11	0,92
B2	10	0,83
C1	9	0,75
C2	8	0,67
C3	7	0,58
D1	6	0,50
D2	5	0,42
D3	4	0,33
E1	3	0,25
E2	2	0,17
E3	1	0,08

### Standardization and weighting

Each indicator has different units and scales that need to be standardized to have comparable in the range 0–100. The formula for standardization is detailed as follows;

$$x=100\;\left(\frac{actual\;value-minimum\;value}{maximum\;value-minimum\;value}\right)=\frac{100\;(x-a)}{b-a}$$ (1)

Hereafter, the standardized indicators were given specific weights based on their significance to the food vulnerability. The weight for each indicator in Table 2 is determined using expert’s judgement. The experts assign weights based on the importance of each indicator by considering factors such as the relevance, significance, or impact of each indicator on the overall analysis or assessment. Hence, the weighting was performed using the rank sum (RS) method and variant methods. In the RS method, the weights correspond to the normalization of individual ranks achieved by dividing each rank by the sum of all ranks. Formula producing the weight is the following [51]:

$${Ra}_{j}=\frac{n-r_{j}+1}{\sum \left(n-r_{j}+1\right)}$$ (2)

Where:

Ra_j_ is the weight of rank, n is number of indicators, and rj is ranking of indicators, j = 1,2,3…n

The variant method was used to weight the value based on the distribution of the data using the following equation:

Vaj=cvari(xij) (3)

Wherec=⌊∑j=1j=k1vari(xij)⌋−1 (4)

Va_j_ = the weight of variance; c = constant; var_i_ = variance of indicator_i;_ xi_j_ = data of indicator _i_

Weighting of SEI/ACI by integrating the two weights using the following equation

w=0.6(Ra)+0.4(Va) (5)

### Calculation of ACI and SEI

The vulnerability of food farming is a function of the ACI and the SEI were calculated using the equation below:

$${ACI}_{i}=\sum_{j=1}^{5}w_{ij}\times I_{Aij}$$ (6)

$${SEI}_{i}=\sum_{j=1}^{5}w_{ij}\times I_{Bij}$$ (7)

Where: w = weight and I: indicator

**Fig 4:**
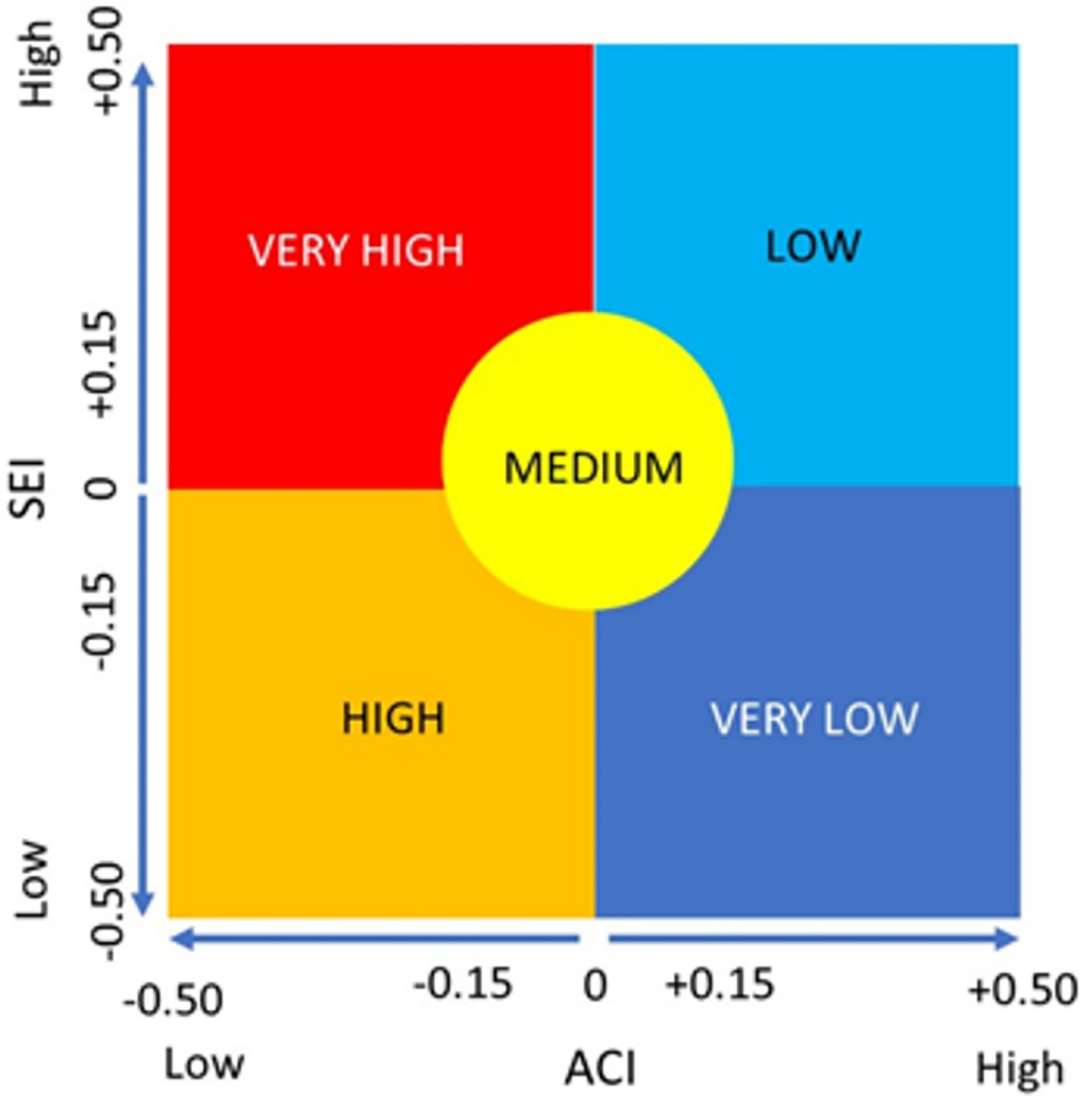
The quadrant system used to rank vulnerability levels. (Source: IPCC 2012, modified).

The vulnerability level was determined by comparing the ACI and the SEI using quadrant methods outlined in Fig 4. The level of adaptation has the opposite effect on the level of exposure and sensitivity to vulnerability. The vulnerability is exacerbated by the high SEI for food farming, but the level of exposure will be further reduced by the higher ACI for food farming. Thus, ACI was drawn on the X axis and SEI on the Y axis.

The ‘Very Low’ group represents areas with the least vulnerability in food farming, indicated by a high ACI value (ranging from 0 to +0.50) and a low SEI value (ranging from -0.5 to 0). The ‘Low’ (light blue) group signifies an area with a low level of vulnerability in food farming, marked by a high ACI value (ranging from 0 to +0.50) but a high SEI value (ranging from 0 to +0.50). The ‘High’ (orange) group is an area with a high level of vulnerability in food farming, indicated by a negative ACI value (ranging from -0.5 to 0) but a negative SEI value (ranging from -0.5 to 0). The ‘Very High’ (red) group is the most vulnerable group and is indicated by a low ACI (ranging from -0.5 to 0) and a high SEI (ranging from 0 to +0.50). The ‘Medium’ (yellow) group is the area in the middle quadrant between the four previous groups with a value ranges between -0.15 to +0.15 for both ACI and SEI.

### Determinant factor

Determinant factors are specific indicators extracted from the ACI and SEI dimensions at a certain threshold value. These indicators are assigned values ranging from 0 to 1, and a threshold of 0.5 is employed to classify an indicator as a determinant factor. The value of 0.5 is considered the middle value and is used as a limit to determine whether there is a need to reduce the level of exposure and sensitivity (SEI) or still need to increase its adaptive capacity (ACI). The ACI cut-off value is set at less than 0.5, which implies that the adaptive capacity is currently insufficient and requires enhancement. Conversely, the threshold for SEI is elevated above 0.5, indicating a higher level of sensitivity and exposure. To reduce the vulnerability in food farming, values that exceed 0.5 should be lowered through specific measures, such as implementing suitable technology and enhancing agricultural infrastructure. Spider graphs are used to visualize and quickly discern areas of strengths and weaknesses for each set of indicators.

### Survey and interview

This survey does not involve an individual respondent as a research subject, and did not ask them any questions relating to their own views, attitudes, concerns, interests, behaviour, achievements or anything else pertaining to them as individuals. The respondents have been informed that the survey result will be used in this research and they have signed consent letter of participation. The vulnerability analysis results were confirmed by interviewing farmers in locations with "very high" and "high" levels of food farming vulnerability. Interviews were conducted only to observe the characteristics of farming related to vulnerability. The selection of the respondents were made based on the discussions with the local agricultural department and the willingness of the farmers to be interviewed, which is reinforced by signing as a respondent.

The survey was carried out in five provinces on the island of Kalimantan in 2018, and within each province, one regency has been selected to represent a “Very High” or “High” level of vulnerability to farming. The five regencies were Sambas (West Kalimantan), Pulang Pisau (Central Kalimantan), Barito Kuala (South Kalimantan), Tanjung Palas (North Kalimantan) and North Panajam Paser (East Kalimantan). In each regency, two subregencies were selected for farmer interviews. The selection of subdistricts involved discussion with the staff of The Office of Agriculture and the Agricultural Extension Centre in each regency.

Respondents were selected based on three age criteria: young (20–40 y.o), adult (41–60 y.o), and senior (60–80 y.o). The age criteria could be subjective in nature, as choice of respondents based on age depended on the researcher. The majority of the respondents are elementary school graduates and are rain-fed rice farmers (74%) having farming experience of 2 to 54 years. Rice is the main food commodity cultivated by respondent farmers (97%). The determination of respondents was based on stratified purposive sampling to collect information through questionnaires which focused on four aspects: (1) General information including the age of farmer, type of agricultural land, commodities, cropping patterns, use of varieties, fertilizers, pesticides and agricultural machinery, (2) Climate-related disasters include floods, droughts, pest and disease attacks, their impacts and farmers’ response to climate-related disasters, (3) Farmers’ capacity including the quantity of production, land ownership area, road access, access to climate information and others, the role of farmer groups, the number of extension workers and government subsidy, and (4) Farmers’ challenges and expectations in implementing farming.

Interviews were conducted by directly asking farmers from the list provided in a questionnaire. To maintain the originality of their responses, the surveyor questioned them individually. Responses were written directly into the questionnaire. To obtain a comprehensive overview of farming characteristics, the responses were compiled and analysed. In total, 150 survey respondents were selected for the interview representing 30 respondents for each regency.

### Digital thematic map

Four thematic maps including Agroclimatic Zone, soil fertility, water criticality and food farming vulnerability, are presented in this paper. Using QGIS, the digital map is generated by overlaying administrative maps of 1:250,000 scale with polygons, for each element of each thematic map.

## Results and discussions

### Climate, land, and water resources of Kalimantan Island

**Fig 5:**
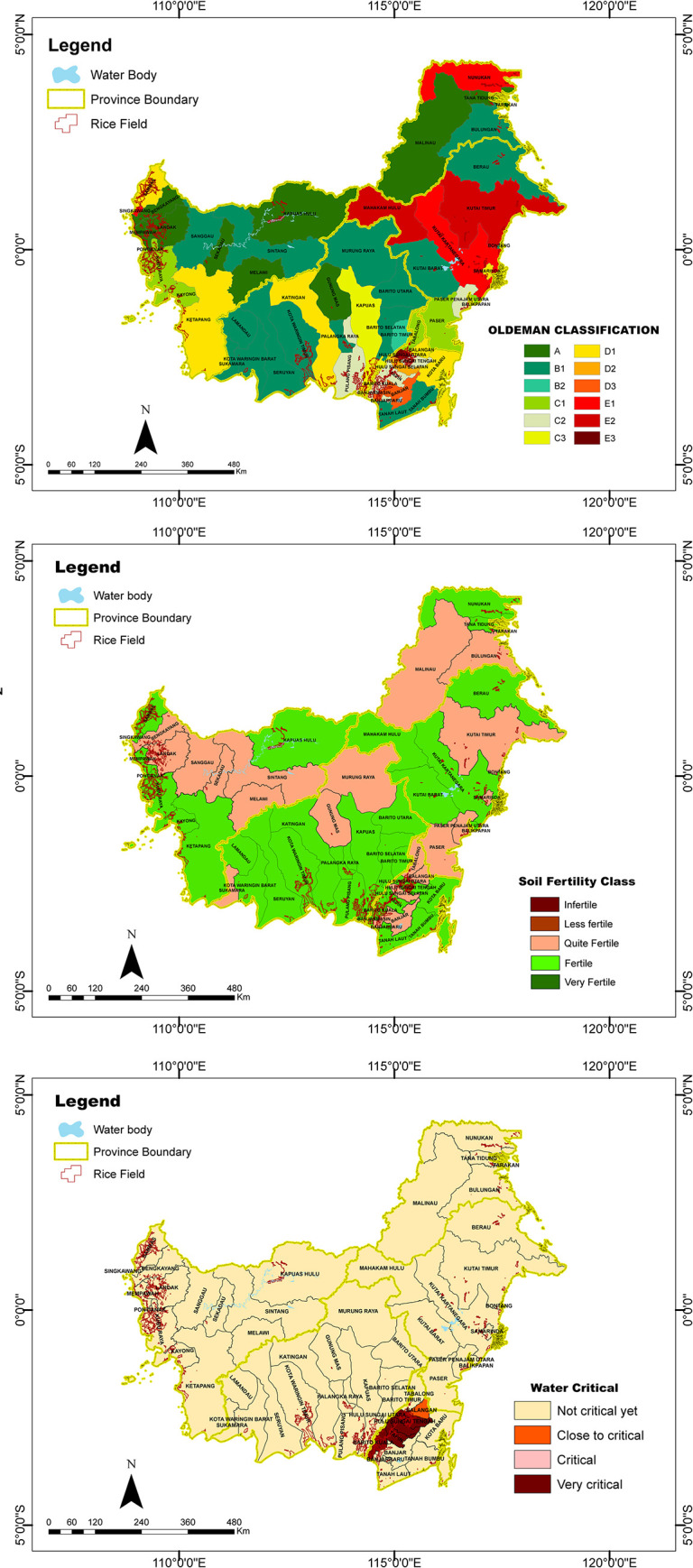
Map of Kalimantan’s (a) agroclimate zone, (b) soil fertility and (c) water criticality.

Food farming is not only influenced by socio-economic factors but also by land resources (climate, soil and water). Kalimantan is predominantly a B1 climate type (30.4% of the 56 regencies/cities) [52]. B1 climate type has seven wet months and no dry months. Other climate types in the region are A (19.6%), C2 (12.5%), D1 (10.7%), C1 (8.9%), E1 (5.4%), E2 (3.6%), and B2, C3, D2, D3, each occupy 1.8%. The driest areas were found primarily in East Kalimantan province, with a small portion found in North Kalimantan. The wettest areas occurred primarily in West and North Kalimantan bordering Malaysia, and parts of Central Kalimantan (Fig 5A). One of the indicators for analysing farming vulnerability, the Climate Index (SEI 12), is the result of identifying this climate type.

The regencies were classified according to their soil fertility score, as very fertile, fertile and moderately fertile. The regency of Hulu Sungai Utara of South Kalimantan has the highest score of 415, placing it in the most fertile class. On the other hand, the regency of Malawi of West Kalimantan has the lowest score of 301, putting it in the moderately fertile class. In terms of soil fertility distribution in the provinces, it appears that the southern region of West Kalimantan province has the most fertile soils, especially in the Sambas regency. Sambas regency is dominated by slopes of 0–8%, with major soil group of Sulfaquents, Fluvaquents, and Endoquaepts with plantations and rice fields land use types. Likewise, the southern region of South Kalimantan and the southwestern region of West Kalimantan, as well as many regents/cities in the East Kalimantan and North Kalimantan, also showed significant presence of fertile soils. These regencies were characterised by low slopes and land suitable for plantations and rice fields (Fig 5B). The soil fertility analysis produces the indicator of soil fertility level (SEI 9).

Water availability on the island of Kalimantan is 583,495.28 MCM (millions of cubic meters), while the water demand is 24,905.90 MCM. Overall, Kalimantan has a water criticality index of 4.3 percent, so it falls within the criteria not critical. At the provincial level, the water criticality index for South Kalimantan, West Kalimantan, Central Kalimantan, East Kalimantan, and North Kalimantan were 46.9, 3.2, 2.7, 1.4, and 0.8%, respectively. These five provinces are therefore classified as not critical. Meanwhile, at the regency level, out of the 56 locations, one regency is classified as close to critical, namely Balangan of South Kalimantan, and six regencies of the same province were classified as very critical, namely Banjarmasin, Barito Kuala, Hulu Sungai Selatan, Hulu Sungai Tengah, Hulu Sungai Utara, and Tapin (Fig 5C). The Water Criticality Index (SEI 8) is used to assess the vulnerability of food farming by using the results of this classification.

### Food farming vulnerability

The severity of the effects caused by extreme and non-extreme weather and climate events is heavily influenced by their exposure and vulnerability levels. Vulnerability and exposure trends play a crucial role in shaping the changes in disaster risk and the resulting impacts [53]. This highlights the importance of identifying the level of vulnerability for the planning, identification and management of impacts to be carried out, including in relation to food farming.

Research in Afghanistan has evaluated the vulnerability profile of smallholder farmers due to climate change using the IPCC Framework. The results showed that most of the smallholder farmers in the hilly zone of the sample districts are highly vulnerable, exposed and sensitive with low adaptive capacity to climate change compared to the plains zone. The high vulnerability in the hilly zone is due to limited resources and low adaptive capacity to cope with disruptions, especially in crop cultivation, in response to climate change [54].

Otto et al. [55] have mapped the social-environmental vulnerability to climate change, and found that the most vulnerable groups to climate change and extreme weather are the poorest and socially marginalized segments of the society. Their vulnerability have greatly influenced by social, demographic, and institutional factors such as gender, age, culture, education, and ethnicity. Our analysis of the evidence shows that gender and age differences in households can lead to significant differences in vulnerability, with women, children, and elderly individuals more prone to suffering. Extreme weather events have a significant impact on the well-being of young children from disadvantaged households, making them particularly vulnerable. Concerns are raised about intergenerational climate justice and the potential for experiencing intergenerational poverty cycles.

**Fig 6:**
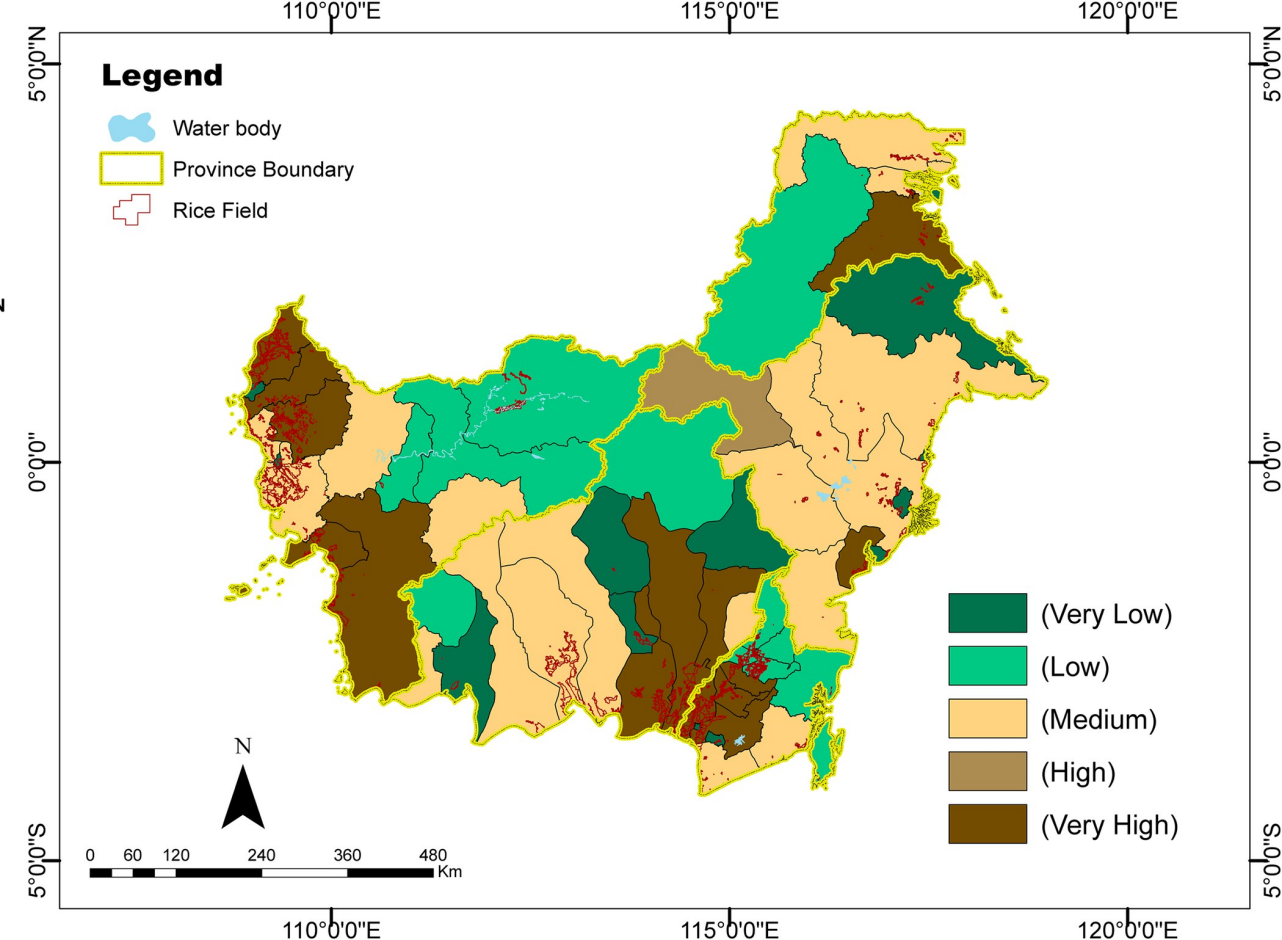
Map of food farming vulnerability in Kalimantan.

The classification of food farming is divided into five vulnerability classes: very low, low, medium, high, and very high (Fig 6). Several regencies have the classification of “very high” and “high” vulnerability, meaning that in these regencies, farming is highly influenced by the dynamics the magnitude of sensitivity, exposure, and adaptive capacity. From the indicated results, 14 regencies/cities are classified as very high level of vulnerability: North Paser Penajam Regency in East Kalimantan Province, Regency of Pulang Pisau, Kapuas, South Barito in Central Kalimantan Province, Bulungan Regency in North Kalimantan Province, Regency of Tapin, Hulu Sungai Selatan, Banjar and Barito Kuala in South Kalimantan Province, and Bengkayang Regency, Kayong, Ketapang, Landak and Sambas in West Kalimantan Province. These regencies are major food producing areas in Kalimantan.

The climatic disasters that are intense in Kalimantan were floods and droughts. Based on the data analysis of floods and droughts by the Directorate of Food Crop Protection for the period 1989 to 2017 (29 years), several regencies of Kalimantan had experienced increased floods and droughts trend [39]. Areas with “very high” and “high” levels of farming vulnerability are areas with an increasing trend of flooding such as North Penajam Paser, Kapuas, South Barito and Mahakam Hulu, while the trend of drought is worsening in North Panajam Paser and Pulang Pisau. In terms of climate, most vulnerable areas have a rainfall type which is sparsely dry (type C to E) where the proportion of dry months is more dominant than wet months. To better understand the factors that most influenced the level of vulnerability in the regencies it is important to identify the determinant factors.

### Determinant factor

The findings of this study indicate that the majority of food farming important to note that 23% of the total 56 regencies are classified as having high to very high levels of vulnerability. The majority of these regencies are important rice-growing areas. The new national capital site of Paser Penajam Utara Regency falls under the category of very high vulnerability.

**Fig 7:**
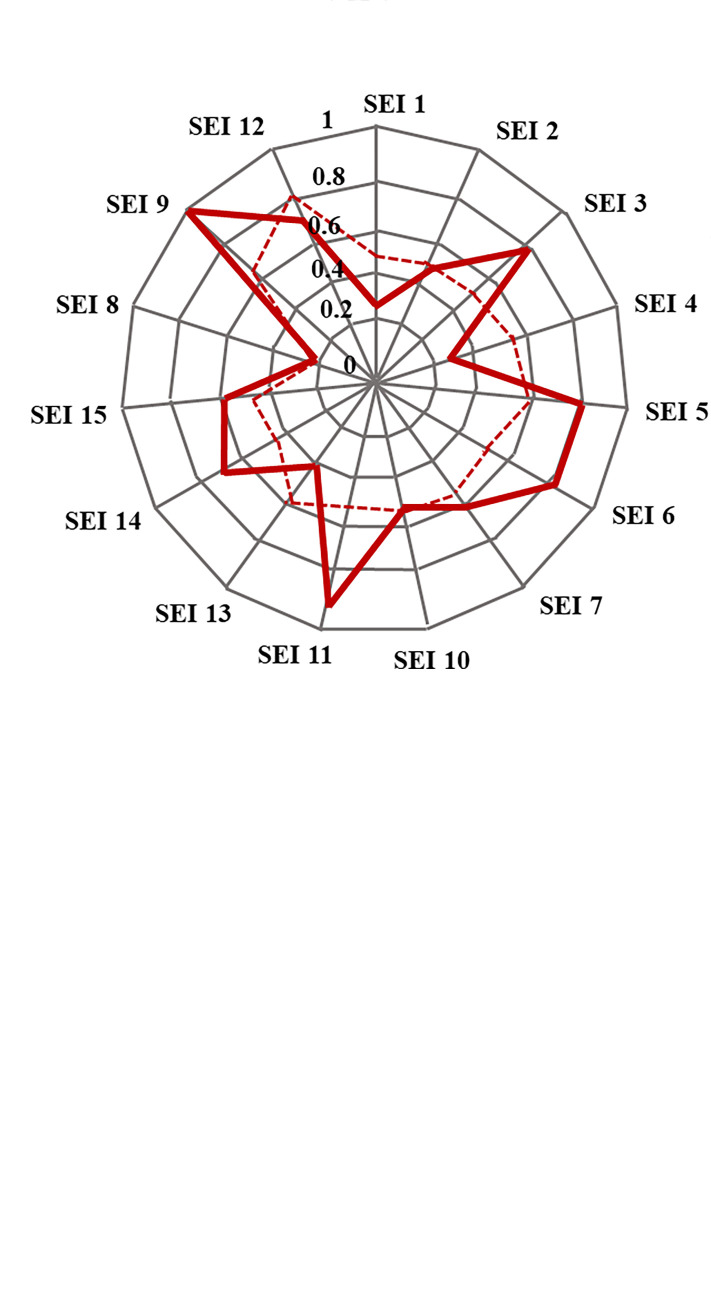
Spider graph showing the relative conditions of the ACI and SEI indicators in the North Paser Penajam Regency of East Kalimantan Province.

The determinant factors of ACI are the ratio of the number of farmers groups per area of rice field (ACI 4), the school participation rate (ACI 1) and the ratio of the number of extension workers per area of rice field (ACI 3). As many as 7 of the 15 indicators from the SEI are determinant factors that require attention as the leverage points for adaptation measures (Fig 7A and 7B). Some of the efforts can be made by improving soil fertility (SEI 9), reducing income gap (SEI 11), increasing food diversification (SEI 3), reducing percentage of poor people (SEI 5), reducing dependence on rice (SEI 6), reducing population density (SEI 14), and increasing agricultural land area (SEI 15). The last two determinant factors will be a challenge to adapt to due to the location of the new country’s capital. Supplies from surrounding regencies are necessary to meet the food needs of the regency.

**Fig 8:**
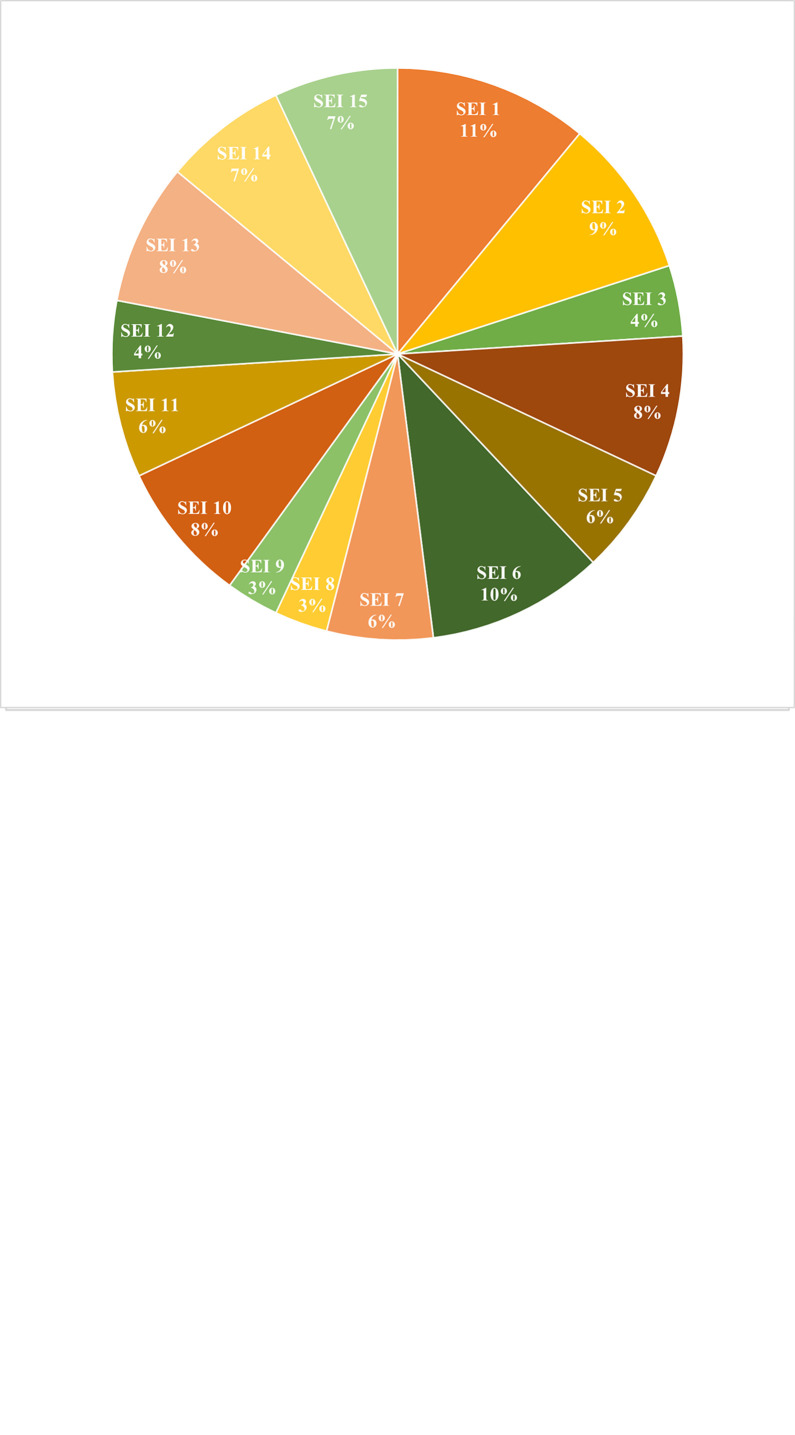
Percentage of ACI (a) and SEI (b) as determinant factors in regencies with a "very high" magnitude of vulnerability to food farming.

Overall for Kalimantan Island, determinant factors contributing to a "very high" vulnerability in agricultural production were identified. The ratio of the number of agricultural machinery to rice area (ACI 5) was the largest contributor (26%), followed by the ratio of the number of farmer groups to rice area (ACI 4), the ratio of the number of extension agents to rice area (ACI 3), school participation rate (ACI 1) and road length based on surface conditions (ACI 2) (Fig 8A). These results indicate the limited availability of agricultural machinery compared to the area of rice fields. In addition, farmer group has a significant impact on adaptability. These groups present a platform for farmers to explore ideas together and experiences related to farming practices such as deciding when to plant and distribute labour and resources [56]. The lack of extension workers also responsible for weak adaptability. Extension workers are also a place to raise adaptation issues related to farming, risks and handling such as the outbreaks of pests and diseases, floods, droughts and other disasters [57]. The educational level of farmers, the management aspect and the farmer’s mindset towards innovation [58, 59]. Road conditions enable farmers to transport seeds, fertilisers, and pesticides etc., and crop yields also affected vulnerability, with rocky and gravel conditions that delay transportation of materials and damaged crops.

The ratio of rice consumption to total carbohydrate intake was greatest for SEI 1 (11%), followed by the ratio of rice and maize production to total population (SEI 6), rice consumption per capita (SEI 2), ratio of rice expenditure to total food expenditure (SEI 4), ratio of agricultural GRDP to total GRDP (SEI 10), ratio of farmer households to total households (SEI 13), population density (SEI 14), ratio of land area for agriculture to total area (SEI 15), proportion of poor people (SEI 5), ratio of soybean production to total population SEI 7, Gini INDEX (income gap SEI 11), ENTROPI (food diversification level SEI 3), Oldeman climate type (SEI 12), category of water availability (SEI 8), soil fertility level (SEI 9) (Fig 8B).

SEI analysis showed that the factors of production, consumption and natural resources have greatest impacts on exposure and sensitivity. The main contributing factor was the consumption of rice to other sources of carbohydrate. In addition, per capita rice consumption remained high due to the significant role of rice as a major staple for the majority of the Indonesian population, and also played important social, economic, and political roles [51]. This high independence on rice led to greater vulnerability. Additionally, the low-income farmer households and those living in poverty were at higher risk.

### Survey and interview

Based on the responses given by the farmers with respect to their views on farming practices and climatic disasters, it can be assumed that all 150 respondents understood the questions. Despite the low educational attainment of the respondents, they understood and responded to the questions. In terms of age, the farmers were mostly categorized as not young and in terms of educational background, they have relatively low level of educational attainment, which was also revealed in other interviews [60] and generally showed the characteristics of farmers in Indonesia [61]. Table 7 describes respondents’ backgrounds.

**Table 7:** Characteristics of survey respondents.

Variable	Number (n = 150)	Percentage
**Gender**		
Male	139	93%
Female	11	7%
**Age**		
20–40 y.o	33	22%
41–60 y.o	92	61%
61–80 y.o	25	17%
**Education**		
No education	3	2%
Elementary school	63	42%
Junior high school	42	28%
Senior high school	36	24%
University	5	4%

**Fig 9:**
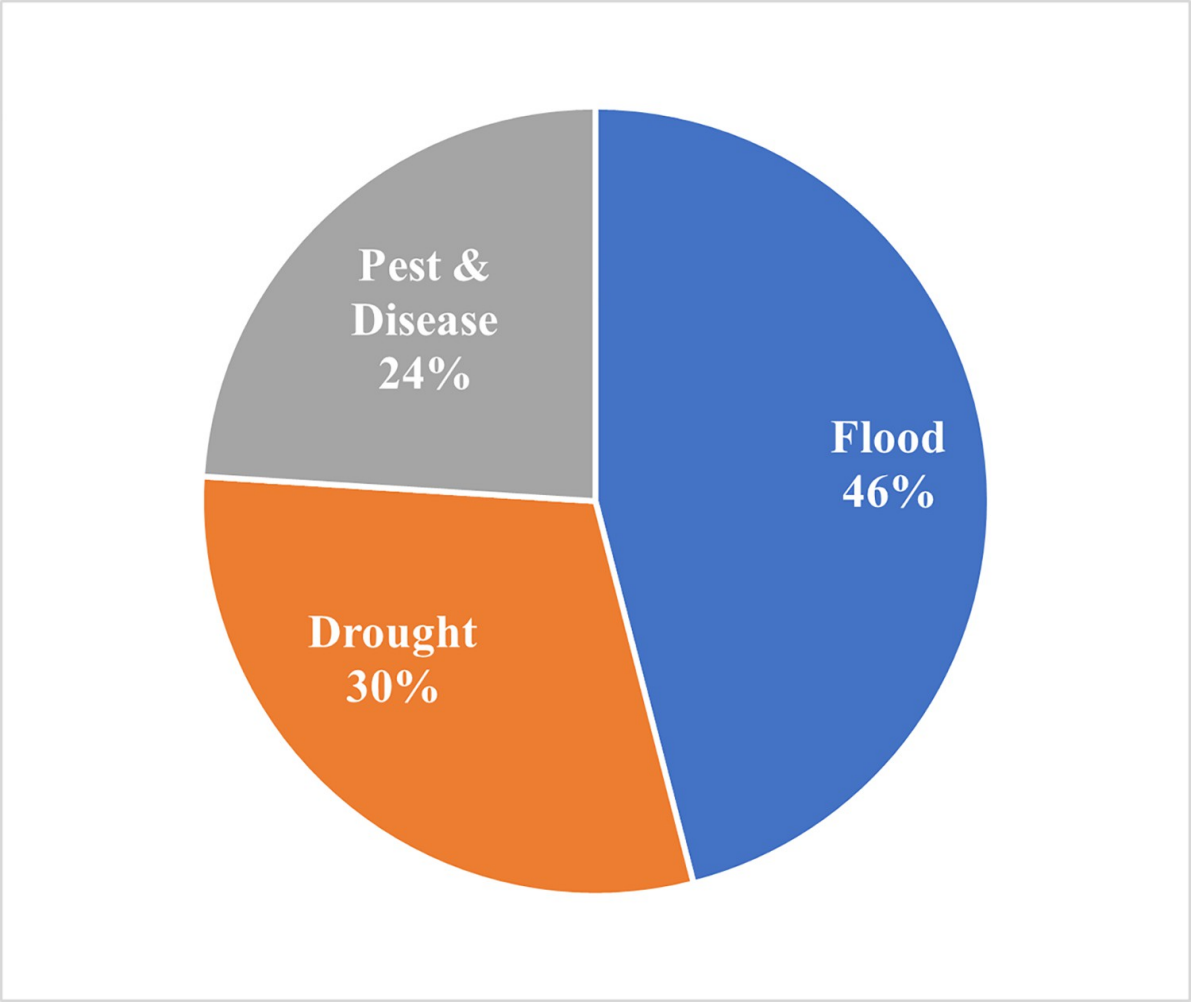
Frequency of climate-related disasters at survey locations for the period 1980–2018.

Of all locations surveyed, 74% were rainfed rice fields and have paddy-paddy cropping patterns (53%). Due to their reliance on rainfall for water availability (54,55), rainfed rice were generally less fertile. The interview revealed that between 1980 and 2018, climate-related disasters occurred on multiple occasions, with floods being the most common disasters (46%), followed by drought (30%) and pests and diseases attacks (24%) (Fig 9).

According to the farmers’ responses, some important points can be made: 1) Road access to their farms comprised dirt roads that became difficult to use during the rain; 2) Farmers found it easy to access information (58%) from sources such as television (47%), extension workers (27%), friends (10%), internet (5%), newspapers (1%) and others (10%); 3) Meeting and consultation with extension workers, for example by providing farmers with advices on pests and diseases management, introducing new planting techniques and varieties, controlling pests and diseases attacks and determining planting and fertiliser schedules as frequent (75%), rarely (21%), and never (4%); 4) The Office of Agriculture provides various aids to help farmers increased production, such as seeds, fertilisers, agricultural machinery, rehabilitation of tertiary irrigation network, rice seed warehouse, drying floor, and demonstration plot.

Based on the interview, a number of challenges faced by the farmers are: first, high pests and diseases attacks, and poor seed quality and availability, which could lead to lower crop yields and ultimately lower farmer’s incomes and could further exacerbate the capital problem for operational costs and reduce access to high quality inputs, creating a negative feedback loop that further decreased productivity and income; second, poor infrastructure, erratic climate and lack of water/irrigation may prevent farmers from getting their products to market, reduced yield, and even led to crop failure; and third, the low rice harvest selling price and the high price of herbicides and pesticides made it difficult for farmers to make profits.

Several recommended programmes to overcome the farmers’ challenges and meeting their expectations for improvements are: 1) Increase the availability of high quality inputs of pesticides, fertilisers, lime, certified seeds, 2) Infrastructure improvements such as improved road, access to water and irrigation, improved storage facilities for marketing crops and reduced crop failures due to extreme climate, 3) Address pest and disease problems such as mass eradication programmes to help farmers reduce damaged crops and enable two planting periods, 4) Stabilise prices by creating a programme that guarantees minimum price and provides price support during low market price, 5) Provide information on climate and weather, capacity building for farmer through climate field school to improve their skills and knowledge.

### Adaptation measures

Countries and communities must develop adaptation solutions and put measures into practice to mitigate the impacts of climate change. Hence adaptation is an indispensable measure in agriculture [56, 57]. Climate change impacts are regionally specific [62]. Consequently, planning, management and adaptation measures need to be taken in response to the level of vulnerability [63].

Regencies with "Very High" and "High" vulnerability can be distinguished by identifying the determinant factors. By examining these determinants, it is possible to identify which factor has the greatest impact of farming in particular regency. As an illustration, in the Panajam Paser Utara Regency of East Kalimantan province, the most crucial SEI is the soil fertility (SEI 9), which holds the lowest score. SEI 9 reflects the capacity of land in an area that supports the growth and production of food crops, especially rice. To enhance SEI 9, recommendations include the implementation of balanced fertilisation, cultivation of rice varieties tolerant to drought and flooding, or soil and water conservation. The next determinant factor is the Gini index or income gap (SEI 11), which indicates the level of inequality. A wider income gap increases vulnerability to disturbances or disasters. The proposed adaptation measures include income distribution, employment opportunities creation, targeted social assistance program and cross subsidies. Based on the SEI and ACI determinant factors, adaptation programs and measures can be identified sequentially as detailed as an example for North Penajam Paser Distrct in Tables 8 and 9. Using the same approach, all regencies in Kalimantan can identify their determinants and propose adaptation measures.

**Table 8:** Determinant factors (SEI) of proposed recommendations in the North Penajam Paser Regency of East Kalimantan.

Index	Indicator	Description	Recommendation
SEI 9	The level of soil fertility	Describes the state of the land in an area that supports the growth and production of food crops, especially rice. Here, soil fertility is estimated based on two aspects, namely soil type and slope. The more fertile the soil, the greater its resistance to disturbances or disasters.	• Implement site-specific balanced fertilisation (and use of organic materials/ fertilisers) • Use of rice varieties tolerant of drought/floods/pests and diseases • Implement land and water conservation management technology
SEI 11	Gini index (income gap)	Describe the level of income inequity in a region. The greater the difference, the easier it is to be exposed to a disturbance or disaster.	• Distribute income • Employment opportunities • Selective social assistance/selective subsidy • Cross subsidies
SEI 3	Entropy	Entropy is a measure of food diversification. This indicator describes the range of dietary sources of carbohydrates consumed by the community. The greater the entropy, the greater the resistance to shock/ disturbance/disaster.	• Enhance food diversification • Develop non-rice local food • Implement sustainable food space (using homegarden to grow vegetables, medicinal plants, fruits, poultry as a source of protein, etc.)
SEI 5	Percentage of poor people	The poor are the ones incapable of providing for their daily needs. This index describes the capacity of a region’s community to respond to their daily needs. The higher the proportion of poor in an area, the greater the sensitivity to disturbances or other disasters.	• Empower community • Social assistance (subsidy selectively specifically for poor farmers/smallholders) • Employment opportunities • Develop infrastructure
SEI 6	Ratio of rice and maize production/ population	Describes the availability of rice and corn-derived foods. The bigger the ratio, the more sensitive it is if there is a disturbance/shock/disaster.	• Enhancement of rice and maize production • Improve/diversify cropping patterns of food crops
SEI 14	Population density	Describes the average per capita area usage rate and presence of farmers in an area. In disruption in food production, densely populated areas and the number of agricultural households, will be more easily exposed.	• Transmigration in synergy with the application of the agrarian reform program • Enhance the implementation of Law No. 41/29 on the protection of sustainable food agricultural land (P2LB)
SEI 15	Ratio of land area for food agriculture to area	Describes the extent to which farmland is occupied and the production of an area. The higher the value, the higher the exposure level for shock/ disruption.	• Create new farmland • Transmigration in synergy with the application of the agrarian reform program

**Table 9:** Determinant factors (ACI) of proposed recommendations in the North Penajam Paser Regency of East Kalimantan.

Index	Indicator	Description	Recommendation
ACI 4	Ratio of number of farmer groups/rice field area	Describes the availability of field facilitators who contribute significantly in helping the community to conduct economic activities and resolve issues. Farmer groups serve as a forum for communication and coordination among farmers to improve understanding and skills. Therefore, areas with a high ratio of the amount of farmer groups to the area of rice paddy fields, if exposed to disturbances or shocks, will be more adaptable.	• Increase the number of farmer groups/Farmer group association • Empower farmer groups to increase capacity building through technical guidance, training, etc.
ACI 1	School participation rate	Describes the of educational level of the local community (elementary school, junior high school, senior high school) with respect to the ability to adapt to possible disturbances or risks. The higher the education, the greater capacity for adaptation.	• Implement 12 years of compulsory education program • Equal sharing of educational amenities up to the village level • Enrich the curriculum related to the technical aspects of adaptation
ACI 3	Ratio of the number of extension workers/rice field area	Describes the availability of field facilitators who play an critical function in helping the community to conduct economic activities and resolve issues. Extension workers are people with a higher level of understanding and capacity in agriculture. Therefore, if the case of disturbances or shocks, areas with a high ratio of the quantity of extension workers to rice area, will have better adaptability.	• Improve extension institutions • Coach, advocate and supervise and improve prosperity to improve the work ethic of extension workers

Surveys and interviews conducted in regencies with "Very High" and "High" levels of vulnerability indicates that these regions have limited natural resources and human resource capacity. Food farming in this area is very vulnerable due to the predominantly low level of education (elementary school), the very small land ownership (on average less than 1 ha), the majority of older individuals (> 50 years old) and the limited infrastructure and facilities. Adaptation to reduce food farming losses can be made using the adaptation measures exemplified in Tables 8 and 9.

## Conclusions

The Island of Kalimantan is expected to develop rapidly with the relocation of Indonesia’s capital to East Kalimantan province. This relocation will require the support of neighboring districts within the Kalimantan provinces to supply food for the new capital. Identifying the drivers that contribute to the susceptibility of food farming on Kalimantan Island is crucial.

The analysis identified 14 regencies of Kalimantan as having “Very High” and “High” vulnerability including North Paser Penajam (East Kalimantan), Pulang Pisau, Kapuas, South Barito (Central Kalimantan), Bulungan (North Kalimantan), Tapin, Hulu Sungai Selatan, Banjar and Barito Kuala (South Kalimantan), and Bengkayang, Kayong, Ketapang, Landak and Sambas (West Kalimantan). These regencies are critical food production areas, and their vulnerability has important implications for food production, supply and security on Kalimantan Island.

Determinants in regions with "Very High" vulnerability to food farming were identified through ACI and SEI indicators. The ACI determinant factor that contributed the most to the level of vulnerability, was the ratio between the number of agricultural machinery to rice field area, contributing about 25.6%, followed by the ratio of the number of farmer groups to rice field area, the ratio of the number of extension agents to rice field area, school participation rate and road length based on surface conditions. In contrast, the most contributing SEI determinant factor were the ratio of rice consumption to total carbohydrate intake (11%) and the ratio of rice and maize production to the total population (SEI 6) (10%). This information was crucial in the formulation of adaptation measures to increase the resilience of food farming, particularly by supporting the new capital of East Kalimantan.

In terms of extreme climate events, the most frequent disasters were floods (46%), drought (30%) and pest and disease attacks (24%), with farmers experiencing strong impacts (49%). Farmers’ responses to the impacts of extreme climate vary widely, with some taking measures such as countering pest attacks, floods, and droughts. However, others remain inactive and waiting for assistance due to limited resources, funds, and technologies, which severely affect their ability to adapt. Several recommended programs to overcome the challenges faced by farmers, including improving infrastructure, increasing the availability of high quality farming inputs, discussing the issues of pests and diseases, and building capacity to understand climate and weather information.

It is expected that the findings of this study can enhance the methodology to measure vulnerability levels of food farming, specifically for farming activities in tropical regions. This study contributes to knowledge and understanding regarding the variability of the study area and agricultural system, farmer characteristics, and agroecosystems for food farming development. Adaptation recommendations are tailored to the location. Future studies can use a similar approach that is tailored to the available and accessible data sources to determine the appropriate adaptation options. Future research can enhance the indicators used to assess food farming vulnerability for other food commodities, horticulture, and plantations grown in various agroecosystems.

## Supporting information

S1 Dataset (XLSX)

S2 Dataset (XLSX)

S1 File (PDF)
