# Supplementary material for: Analysing food farming vulnerability in Kalimantan, Indonesia: Determinant factors and adaptation measures
Source: PLoS One. 2024 Jan 3;19(1):e0296262. doi: 10.1371/journal.pone.0296262 (PMC10763963; doi:10.1371/journal.pone.0296262)
Supplement: S1 File — (PDF) [file pone.0296262.s003.pdf]

KUISIONER KERENTANAN USAHATANI PANGAN DAN RISIKO IKLIM

MENDUKUNG ADAPTASI TERHADAP PERUBAHAN IKLIM

(QUESTIONAIRE OF FOOD FARMING VULNERABILITY AND CLIMATE RISK TO SUPPORT CLIMATE CHANGE ADAPTATION)

1. Informasi Umum (*General Information*)

- Nama Enumerator (*Enumerator's Name*)
  Nama Depan: ……………………….. Nama Belakang: ………………………….
  (*First Name*) (*Last Name*)
- Nama Responden (Respondent's Name)
  Nama Depan: ……………………….. Nama Belakang: ………………………….
  (*First Name*) (*Last Name*)
- Demografi (*Demography*)
  Usia (*Age*) : ……………………
  Pendidikan (*Education*) : ……………………
- Jumlah tanggungan dalam keluarga : …….. person
  (*Number of dependent family members*)
- Lamanya Bertani (*Farming experience*): …… years
- Tipe lahan pertanian (*Type of farmed land*):
  ☐ Irigasi *(Irrigated*) ☐ Tadah hujan (*Rainfed*) ☐ Lainnya : ………………….
  (*Others*)
- Komoditas pertanian (*Agricultural commodities*):
  ☐ Padi (*Rice*) ☐ Jagung (*Maize)* ☐ Kedelai (*Soybean*) ☐ Palawija (*secondary crops*) ☐ Lainnya : ……… (*Others*)
- Pola tanam (*Cropping pattern*):

  ……………………………….. - ………………………………. - ……………………………
- Penggunaan saprotan (*Use of agricultural technologies*)
  Jenis Varietas (*Crop variety*) : …………………………………………………………
  Jenis pupuk (*Fertilizer Type*) : …………………………………………………………
  Jenis pestisida (*Pesticide Type*): …………………………………………………………
  Penggunaan alsintan mulai dari olah tanah sampai pasca panen (*Use of agricultural machinery from soil tillage until post-harvest*):
  ……………………………………………………………………………………………………
  Status Alsintan (*Status of agricultural machinery*): ☐ Sewa (*Renting*) ☐ Milik Sendiri (*Owned*)

2. Verifikasi Bahaya dan Potensi Dampak Iklim Terhadap Pertanian (*Verification of Danger and Potential Climate Impact to Agriculture)*

- Hama dan Penyakit Tanaman pada Musim Tertentu (*Pest and Diseases in Certain Season)*

  MH / *Rainy Season* : ……………………………………………………………………

  MK / *Dry Season* : ……………………………………………………………………

- Apakah Pernah Terjadi Bencana Terkait Iklim (Is there any climate related disasters)?

  ☐ Ya (*Yes*) ☐ Tidak (*No*)

- Contoh Bencana (Example of disasters):

  ☐ Banjir *(Flood)* ☐ Kekeringan *(Drought)* ☐ OPT *(Pest)*, Tahun: ……… *(Year)* Dampak: *(Impact)* ☐ Kuat, *(Strong)* ☐ Sedang *(Medium)* ☐ Ringan *(Weak)*

- Waktu Terakhir Terjadi Bencana (*Most recent occasions of disaster*):
  - Banjir (*Flood*) Tahun: (*Year*) ………..… Bulan: (*Month*) ……………… Lama Kejadian: (*Duration*) ………………………
  - Kekeringan, (*Drought*) Tahun: (*Year*) ………..… Bulan: (*Month*) ……………… Lama Kejadian: (*Duration*) ………………………
  - OPT, (*Pests*) Tahun: (*Year*) ………..… Bulan: (*Month*) ……………… Lama Kejadian: (*Duration*) ………………………

- Mengapa Bencana terjadi? (*Cause of disaster*)

  Banjir (*Flood*):

  ……………………………………………………………………………………………………

  Kekeringan (*Drought*):

  ……………………………………………………………………………………………………

  OPT (*Pests*):

  ……………………………………………………………………………………………………

- Faktor Khusus (*Special factor*):

  ☐ Pendangkalan Sungai (*River sedimentation*) ☐ Perubahan Lahan (*Land-use change*)

  ☐ Wilayah Cekungan (*Basin area*) ☐ Lainnya (*Others*): ……………………………………..

- Dampak Bencana Banjir Terhadap Pertanian (*Impact of Flood to Agriculture*):

  ……………………………………………………………………………………………………

  ……………………………………………………………………………………………………

- Dampak Bencana Kekeringan Terhadap Pertanian (*Impact of Drought to Agriculture*):

  ……………………………………………………………………………………………………

  ……………………………………………………………………………………………………

- Dampak Bencana OPT Terhadap Pertanian – Selain Banjir dan Kekeringan (*Impact of Pests and Diseases to Agriculture – Other than Flood and Drought*):

  ………

  ………

- Dampak Bencana Lainnya (*Impact of Other Disasters*):

  ………

  ………

- Kerugian yang ditimbulkan (*Losses incurred*):

	Waktu Tanam *(Planting Time)*	Produksi *(Production)*	Varietas *(Variety)*	Pemupukan *(Fertilizer Application)*	Luas Panen *(Harvested Area)*
Banjir (*Flood*)	☐	☐	☐	☐	☐
Kekeringan (Drought)	☐	☐	☐	☐	☐
OPT (Pest and Diseases)	☐	☐	☐	☐	☐
Lainnya (Others)	☐	☐	☐	☐	☐

- Respon Petani Terhadap Bencana Terkait Iklim (*Farmer's response towards climate-related disasters*)

Banjir (*Flood*):

………

Kekeringan (*Drought*):

………

OPT (*Pests*):

………

3. **Verifikasi Karakteristik Iklim terkait Kejadian Bencana** (*Verification of Climate Characteristics Associated with Disasters Occurrence*)
   - Ketersediaan air melalui Sungai atau Irigasi (*Water availability from River or Irrigation*):

………

………

   - Perubahan ketersediaan air untuk lahan pertanian selama 5 tahun (*Changes in water availability for agricultural land over 5 years*):

  ………

  ………

- Karakteristik Curah Hujan (*Rainy season characteristics*)

Periode MH (*Rainy season*) : ……………………………MK (*Dry season*) :………………………………..

Bulan puncak MH (*Peak Rainy Season*) : ……………………MK (*Peak Dry Season*): …………………..

Perubahan MH (*Changes of Rainy Season*) : ……………………………………………….. ………… MK (*Changes of Dry Season*): ………………………………………………

4. **Verifikasi Kerentanan dan Risiko Iklim Terhadap Pertanian** (*Verification of Climate Risk and Vulnerability Towards Agricultur*e)
   - KAPASITAS (*CAPACITY*)

- Besar Produksi / Produktvitas Padi (*Rice production or productivity)*:

………

- Luas kepemilikan lahan pertanian *(Area of agricultural land ownership)*:

………

- Deskripsi kondisi lahan termasuk kesuburan dan kemiringan tanah *(Description of land conditions including soil fertility and slope*):

……

……

……

- Akses dan jenis jalan (*Access and type of road*):

………

………

- Akses terhadap informasi iklim dan lainnya serta sumber informasi (*Access to climate and other informations with information source*): ☐ Mudah (*Easy*) ☐ Sulit (*Difficult*)
  Sumber (Sources): ☐ TV/Radio ☐ Koran (*Newspaper*) ☐ Penyuluh (*Agriculture Extension Officer*) ☐Teman (*Friend*) ☐Internet ☐ Lainnya (*Others*): …………..
- Jumlah Poktan (*Number of farmers group*)
- Keterlibatan dalam Poktan (*Involvement in Farmers Group*) : (Ya/*Yes* or Tidak/*No*) Jika Ya, Sebagai (If *Yes, details the role*): …………………………………………

Peran Poktan dalam mendukung usaha tani (*Farmers group role in supporting farming activities*):

……

……

……

- Jumlah penyuluh (*Number of Agriculture Extension Officer*): ………………………………………
- Aktivitas penyuluhan (*Extension activities*): (Sering / Jarang / Tidak) Contohnya :
  (Frequent / Scarce / No Activities), for example:

……

……

- Adakah Pengolahan Hasil Panen (*Is there any post-harvest processing*)?
  Ya/Tidak (*Yes/No*). Jelaskan (*Please detail*): ……………………………………………………
  ……………………………………………………………………………………………………
- Bantuan/pinjaman Modal (*Capital grant / loan*):
  ☐ Pemerintah (*Government*) ☐ Lembaga (*Institution*) ☐ Bank ☐ Keluarga (*Family*)
  ☐ Lainnya (*Others*):……..
- Keikutsertaan dalam Asuransi Pertanian (*Participation in Agricultural Insurance*):
  ☐ Sudah (*Already participated*) ☐ Belum (*Not yet*) ☐ Tidak (*Not Participating*).
  Alasan (*Reason*): ……………………………………………………………………………
- Bagaimana harga jual hasil pertanian di MH dan MK (*Please detail the sales of the agricultural products in rainy and dry season*)?
  MH (*Rainy season*) : ……………………………………………………………………………
  MK (Dry season) : ……………………………………………………………………………
- Distribusi Hasil Pertanian (Agriculture products distribution):
  ☐ Tengkulak (*Middlemen*) ☐ Pasar (*Traditional Market*) ☐ Koperasi (*Cooperative organization*) ☐ Poktan (*Farmer's Group*) ☐ Lainnya (*Others*):………………………………
- Bentuk Bantuan Peningkatan Produksi Pertanian dari Pemerintah/Poktan (*Forms of Assistance for Increasing Agricultural Production from the Government/Farmers Group*):

……

……

……

- KONSUMSI (*CONSUMPTION*)

- Komoditas pangan utama untuk konsumsi harian (selain beras) :
  Main food commodities for daily consumption (other than rice)

……

……

………

- Jumlah pengeluaran untuk konsumsi beras dan non-beras sebagai konsumsi utama :
  Total of expenditure for rice and non-rice consumption as main food consumption

………

………

………

- Akses terhadap bahan pangan pada saat kejadian bencana :
  Access to food produce in times of disasters

………

………

………

- KENDALA DAN HARAPAN (*CONSTRAINTS AND EXPECTATIONS*)

- Kendala utama dalam kegiatan usaha tani:
  Main constraints in food farming activities

………

………

………

- Harapan dalam kegiatan usaha tani :
  Expectations in food farming activities

………

………

………
